# Quantitative Investigation of the Link between Actin Cytoskeleton Dynamics and Cellular Behavior

**DOI:** 10.3390/mi13111885

**Published:** 2022-11-01

**Authors:** Ying Li, Xiaoru Zhuang, Fuzhou Niu

**Affiliations:** 1 Department of Mechanical and Electrical Engineering, Shenzhen Polytechnic, Shenzhen 518055, China; 2 School of Mechanical Engineering, Suzhou University of Science and Technology, Suzhou 215000, China

**Keywords:** mechanobiological, actin cytoskeleton, optical tweezers, β-actin

## Abstract

Actin cytoskeleton reorganization, which is governed by actin-associated proteins, has a close relationship with the change of cell biological behavior. However, a perceived understanding of how actin mechanical property links to cell biological property remains unclear. This paper reports a label-free biomarker to indicate this interrelationship by using the actin cytoskeleton model and optical tweezers (OT) manipulation technology. Both biophysical and biochemical methods were employed, respectively, as stimuli for two case studies. By comparing the mechanical and biological experiment results of the leukemia cells under electrical field exposure and human mesenchymal stem cells (hMSC) under adipogenesis differentiation, we concluded that β-actin can function as an indicator in characterizing the alteration of cellular biological behavior during the change of actin cytoskeleton mechanical property. This study demonstrated an effective way to probe a quantitative understanding of how actin cytoskeleton reorganization reflects the interrelation between cell mechanical property and cell biological behavior.

## 1. Introduction

The actin cytoskeleton governs the mechanical properties of cells to a large extent and mediates a variety of essential cellular biological characteristics in all eukaryotic cells through structural alteration [1,2]. The interrelationship between cell biological behavior including fusion, migration, adhesion, differentiation, proliferation, apoptosis, etc, and actin cytoskeleton reorganization, has been discussed in numerous research works [3,4,5,6,7,8]. Gardel et al. found that the surface topography profoundly influences cell adhesion, differentiation, and stem cell fate control [3]. Ménasché et al. demonstrated that the FcεRI-mediated stimulation in rat basophilic leukemia (RBL) MC promotes a decrease in cellular F-actin content during the first 10–30 s followed by a rapid increase within 1 min [4]. Wang et al. utilized nanofiber-orchestrated cytoskeletal reorganization to promote the migration speed of both collective multicellular strands and single cells [5]. Uetaki et al. found that the depolymerization of the actin cytoskeleton triggers the differentiation of preadipocytes into mature adipocytes [6]. Fan reviewed how the remodeling of actin filaments occurs under static mechanical force eventually affects cell proliferation and differentiation [7]. Mo et al. provided evidence to confirm that disrupting the formation of actin polymers leads to osteoblast apoptosis [8].

Cell stiffness is strongly affected by the microenvironment in which cells live. Cells respond to biochemical and biophysical cues through alterations in morphology, stiffness, and gene expression profiles, or the structural rearrangement of the cytoskeleton itself [9,10]. Various cases of biochemical and biophysical stimuli on cytoskeleton have been introduced to emphasize the interrelationship between the actin dynamics and biological behavior of single cells. The mechanism of how biochemical stimuli affect the actin cytoskeleton, for example, drug-induced cellular differentiation, was widely investigated [11,12,13,14]. As one of the typical biophysical stimuli, electric field exposure, gained huge attention in research works [15,16,17,18]. Since a key determinant of cell mechanical properties is the actin cytoskeletal network, changes of cellular stiffness correlate with alteration in actin structure and content. Our previous works have thoroughly discussed the relationship between the mechanical properties of the actin cytoskeleton and the actin reorganization using an actin-based micro-structural model, where the actin cytoskeleton mechanical parameters were extracted as label-free markers of the interrelationship between actin mechanical dynamics and cell biological properties [19,20,21]. However, for further quantitative investigation of the cell mechano-biological interrelationship, a more appropriate indicator is needed to reveal the inner linking.

Among the six actin isoforms that have been identified in humans, β-actin and γ-actin are the most ubiquitously expressed [22]. β-actin is commonly used to normalize molecular expression studies due to its high conservation as an endogenous housekeeping gene. However, recent research showed that β-actin expression can change in response to biochemical stimuli during growth and differentiation, and in disease states [23]. The highly dynamic organization of the actin cytoskeleton is tightly controlled by a variety of actin-related proteins (Arp) that regulate actin nucleation, polymerization, depolymerization, branching, bundling, and localization [24]. The actin-related protein Arp2/3 complex plays a central role in the assembly of actin networks by producing branched filaments to push forward the leading edge of motile cells and anchoring the new filament to the preexisting actin network [25]. Rho family GTPase is also the key regulatory molecule involved in the remodeling of the actin cytoskeleton. About 20 different types of Rho family proteins, including RhoA and Rac1, are essential in actin reorganization [26]. RhoA is mainly responsible for the generation of cell force and tension within the cell. Rac1 is thought to regulate lamellipodia formation and act upstream of RhoA during actin cytoskeleton reorganization [27]. The genes expressions alteration of these proteins associated with actin cytoskeleton indicates the structural reorganization of the actin cytoskeleton, which may have a close relationship with the change of cell biological behavior.

In this work, we proposed a possible indicator of how the cellular biological behavior was linked to actin cytoskeleton dynamics. Electric field exposure and drug-induced differentiation were applied as the biophysical and biochemical stimuli to induce actin cytoskeleton reorganization. The mechanical properties of the actin cytoskeleton were obtained by extracting key parameters from the OT stretching experiment results utilizing the actin microstructural model. The expression of genes related to cell biological behavior was measured through the reverse transcription polymerase chain reaction (RT-PCR). Proteins associated with actin cytoskeleton dynamics were also tested. By comparing the results of two case studies, we concluded that β-actin could act as an indicator to quantitatively characterize the interrelationship between the biological and mechanical properties of single cells.

## 2. Materials and Methods

### 2.1. Cell Culture and Treatment

Adipocyte differentiation: human MSCs (PT-2501, Lonza, Basel, Switzerland) were cultured in a MSC medium bullet kit (PT-3001, Lonza) and maintained in a humidified atmosphere of 5% CO_2_/95% air at 37 °C. For the adipogenesis differentiation experiment, hMSCs were cultured at 4 × 10^3^ cells/cm^2^ 12-well plate until confluence, the medium was then replaced with an adipogenesis induction medium (A10070-01, Gibco, Waltham, MA, USA). Cells maintained in the induction medium for 2, 4, and 6 days were collected for optical stretching operation. The control group cells were cultured without any induction factor.

### 2.2. Electric Stimulation

Leukemia NB4 cells were cultured in RPMI-1640 medium (11875085, Gibco) supplemented with 10% fetal bovine serum (10270106, Gibco) and 100 U/mL penicillin and streptomycin (15240-062, Gibco), in a humidified incubator of 5% CO_2_ and 37 °C environments. NB4 cells and medium were placed in a designed chamber with both sides made from carbon mixed PDMS power as electrodes for electrical stimulation. Different AC voltages of 5, 10, and 20 V were supplied to the chamber by the signal generator at the frequency of 500 kHz.

### 2.3. Optical Stretching

Streptavidin-coated polystyrene beads (Bangs Lab, Fishers, IN, USA) were incubated in phosphate-buffered saline with biotin-conjugated concanavalinA (ConA, Sigma-Aldrich, St. Louis, MO, USA) at room temperate for 30 min. ConA was coated on the beads through avidin-biotin interaction. Then, ConA-coated beads were added to the cell solution and incubated at 37 °C for 30 min. In the presence of Ca^2+^ and Mn^2+^, ConA-coated beads were bonded to the cell membrane and acted as handles for optical manipulation. Stretching manipulation experiments were conducted using the OT system (BioRyx 200; Arryx, Chicago, IL, USA) with the resolution of pN.

The stretching manipulation experiments were conducted using the optical tweezers system (BioRyx 200; Arryx) with the resolution of piconewtons (pN). Beads-bonded cells were loaded into a glass bottom dish, and placed in the microscope stage of the optical tweezers system. A laser beam with a wavelength of 1064 nm was employed to create multiple optical traps. By controlling the position of the optical trap, the beads attached to the cells were moved away from the cell center point. A reaction force was generated to stretch the cells. The stretching force under different laser powers was measured by the viscous drag force calibration method.

### 2.4. Actin Cytoskeleton Microstructural Model

The actin cytoskeleton microstructural model developed in the previous work [28] was utilized to interpret how the actin cytoskeleton dynamics were translated into the alteration of the F-actin structural parameters. The model was represented by a three-dimensional actin cytoskeleton random network with actin-binding proteins (ABPs) randomly distributed.

The MacKintosh-derived WLC model was adopted to describe the semiflexible mechanical behavior of F-actin [29], and ABPs were represented by linear springs.

$$f_{a}=\frac{81k_{b}TL_{p}^{2}L_{c}^{2}(\Delta r+\delta r_{0})}{{\left(L_{c}^{2}-6L_{p}\Delta r-6L_{p}\delta r_{0}\right)}^{2}(L_{c}^{2}+3L_{p}\Delta r+3L_{p}\delta r_{0})}$$

where Δ*r* is the extension of the actin filament, *δr*_0_ is the pre-extension of the actin filament caused by prestress, *L_p_* is the persistence length, *L_c_* is the contour length, *k_b_* is the Boltzmann’s constant, and *T* is the absolute temperature. In addition, the relationship among the contour length (*L_c_*), the diameter of the F-actin (*d_Actin_*), actin concentration (*C_AF_*), and density of the crosslinks (*R*) of the actin network can be expressed as follows:

$$L_{c}=\frac{R^{0.2}d_{Actin}}{2}\sqrt{\frac{\pi }{C_{AF}}}$$

By fitting the modeling results into the experimental data, key mechanical parameters of the actin cytoskeleton can be extracted [21].

### 2.5. RNA Isolation and RT PCR Analysis

The relative quantity of mRNA expression was tested by RT-PCR. The total RNA of the cells was extracted using Trizol reagent (Ambion, Austin, TX, USA). mRNA was reverse-transcribed to cDNA by using iScriptTM cDNA Synthesis Kit (Bio-Rad, Hercules, CA, USA). Quantitative RT-PCR amplification was performed with SsoAdvanced SYBR Green Supermix Kit (Bio-Rad) in the CFX96 System (Bio-Rad). In sample analysis, the threshold was set based on the exponential phase of amplified products, and the cycle threshold for each target gene was determined. The expression of the target gene was normalized to that of the 18 s housekeeping gene.

## 3. Experiments and Results

### 3.1. hMSCs Stretching Experiment

**Figure 1:**
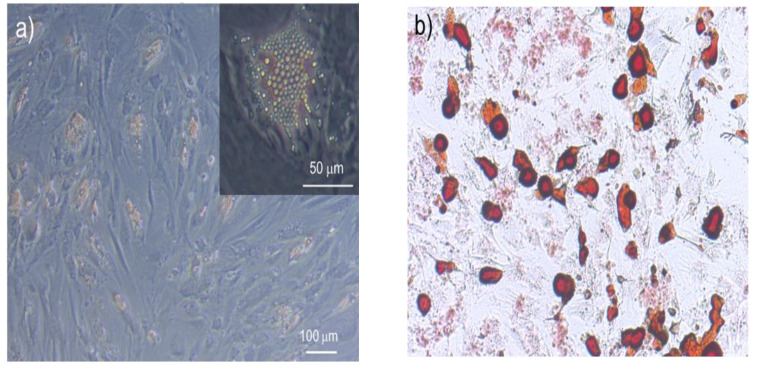
Differentiation of hMSCs into the adipogenic lineage. (**a**) Lipid droplets accumulated in maturated adipocytes. (**b**) Oil red O staining of hMSC-derived adipocyte.

**Figure 2:**
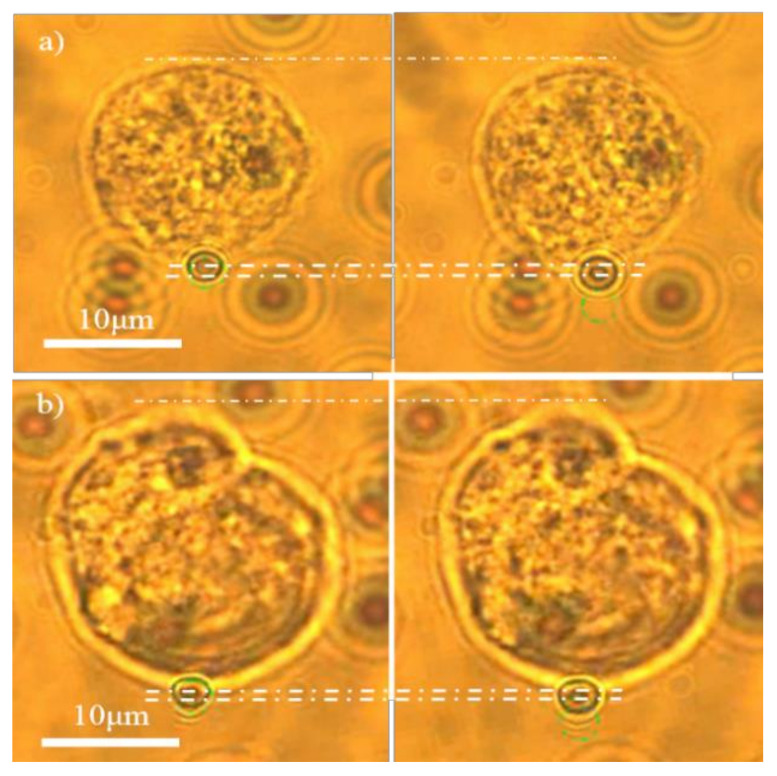
Cell deformation of (**a**) hMSCs and (**b**) hMSC-derived adipocytes under optical stretching.

**Figure 3:**
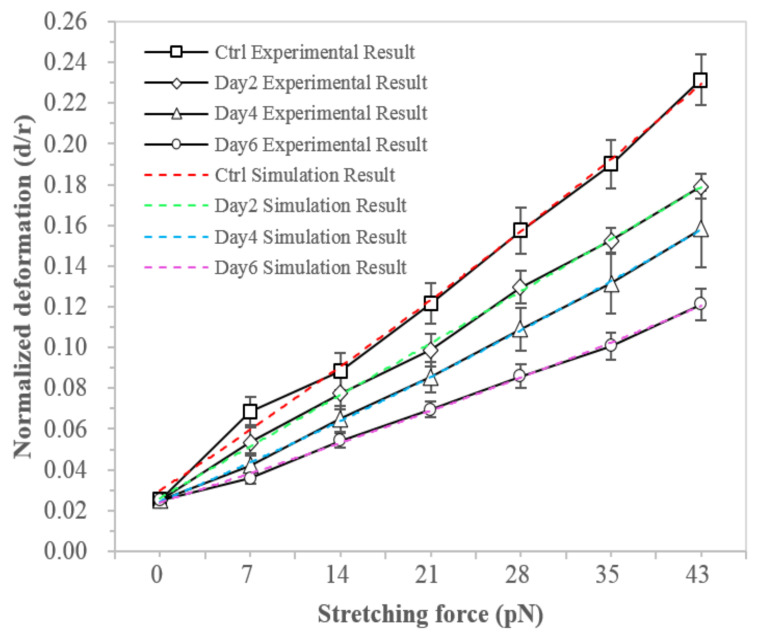
Deformation behavior of hMSCs during adipogenic differentiation.

The differentiation process of MSCs into adipogenic lineage is shown in Figure 1. We firstly examined the change of the hMSCs mechanical properties during adipogenic differentiation. The deformation of hMSCs in both control and differentiated groups in the induction medium for 2, 4, and 6 days were measured by OT stretching experiments, with the process shown in Figure 2. Within the range of the applied stretching forces, the deformation of the differentiated group was smaller than the control group, as shown in Figure 3. These results indicated that the hMSC-derived adipocytes were stiffer than the undifferentiated ones. Under the same stretching force, the deformation of the differentiated cells at the earlier stage, such as day 2 and 4, was larger than those at the later stage like day, which indicated cell stiffness increases during the adipogenic differentiation and maturation process.

The structural parameters of F-actin were extracted by fitting the modeling results into the stretching experiments as shown in Table 1. These structural parameters facilitated the quantification of the underlying F-actin remodeling profile in showing that the mechanical properties of hMSCs correlate with adipogenic differentiation. Figure 3 illustrated that the modeling normalized deformations (deformation under force versus cell radius) of the hMSCs and the hMSC-derived adipocytes matched well with the experimental results.

### 3.2. NB4 Cells Stretching Experiment

**Figure 4:**
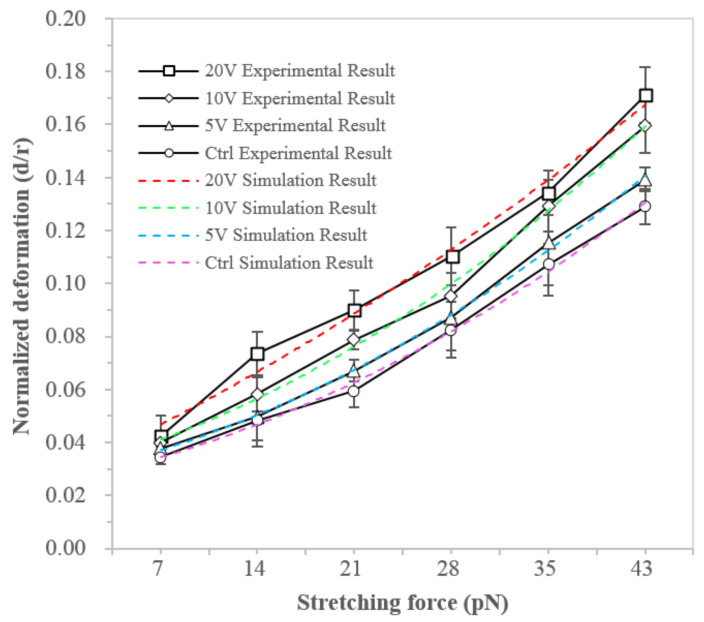
Alteration of the deformation behavior of NB4 cells under electric stimulation.

We further examined the change in the mechanical property of NB4 cells treated by electric stimulation. Through stretching cells with optical tweezers, the stiffness of cells treated by electric stimulation, with voltages of 5 V, 10 V, and 20 V, respectively, were measured and compared with the control group. Figure 4 illustrated the force-deformation relationship obtained by both experimental and modeling studies, where the normalized deformation of electric filed treated cells was larger than the control group under the same stretching force (*p* < 0.01), indicating the decrease of NB4 cell stiffness after electric treatment [30]. The modeling results of NB4 cells have a good match with the experimental results. Through fitting modeling results into the experimental results, the estimated actin cytoskeleton parameters could be extracted, as given in Table 2.

The results of both case studies showed that under external stimuli, either medium-induced adipogenic differentiation or electrical stimulation, could lead to the F-actin reorganization and subsequently affect the cell’s mechanical properties. The mechanical behavior of the F-actin network was determined by the persistence length and density of F-actin and the density of crosslink. These parameters could be extracted by fitting the modeling results into the experimental data. The persistence length of F-actin and the density of crosslink could be adopted from previous work [21]. The number of F-actin in the network was calculated based on the density of F-actin and the external/internal radii of the F-actin network. For the hMSCs, the actin distribution enlarged and widened, and the density of actin increased during differentiation. For NB4 cells, the size of actin distribution decreased and became narrow, and the density of actin decreased after the electrical stimulation Therefore, the number and density of F-actin were closely related to the mechanical stiffening of the two types of cells.

### 3.3. Gene Analysis

We further analyzed gene expression through quantitative RT-PCR to characterize the alteration of biological properties during adipogenesis and electric stimulation treatment. The gene expression levels of β-actin and actin-related proteins including Arp2/3, RhoA, and Rac1, were measured. In the adipogenesis case study, PPARg is tested as the overexpression of PPARg may indicate the adipocyte differentiation [31]. In the electric stimulation case study, the expression of Bcl-2 was tested. Bcl-2 plays an important role in cell apoptosis induced by anticancer agents; Bcl-2 overexpression was reported in many cancers [32].

**Figure 5:**
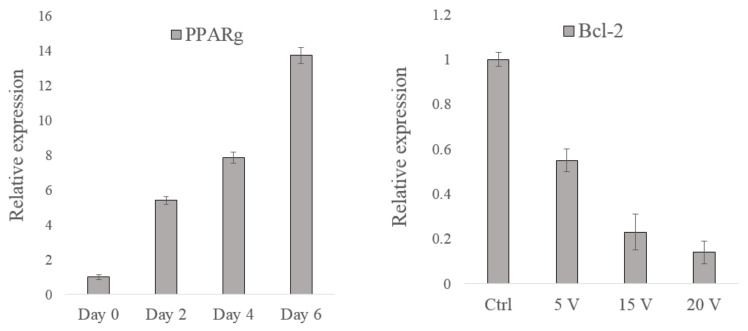
Gene expression results of the alteration of cell biological properties in two case studies. (The (**left**) one is the PPARg expression for hMSC and the (**right**) one is Bcl-2 for NB4 cells).

Figure 5 illustrates the gene expression results of the two case studies. The PPARg expression increased as the time the hMSCs were in the induction medium was prolonged, indicating that the adipocyte differentiation level of the hMSCs was enhanced. The Bcl-2 expression decreased as the applied electric stimulation voltage increased, implying that the cell apoptosis level increased. These results revealed that the biological properties of the cells changed after they were treated.

**Figure 6:**
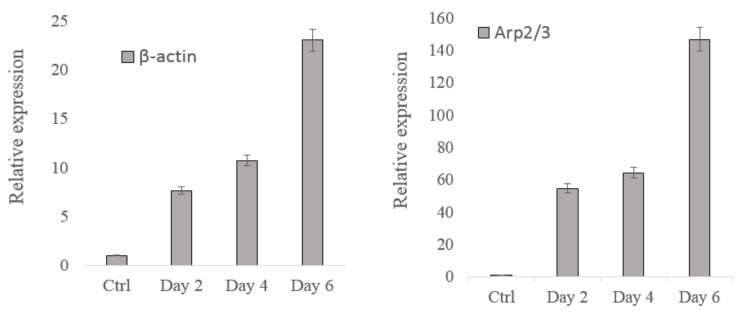

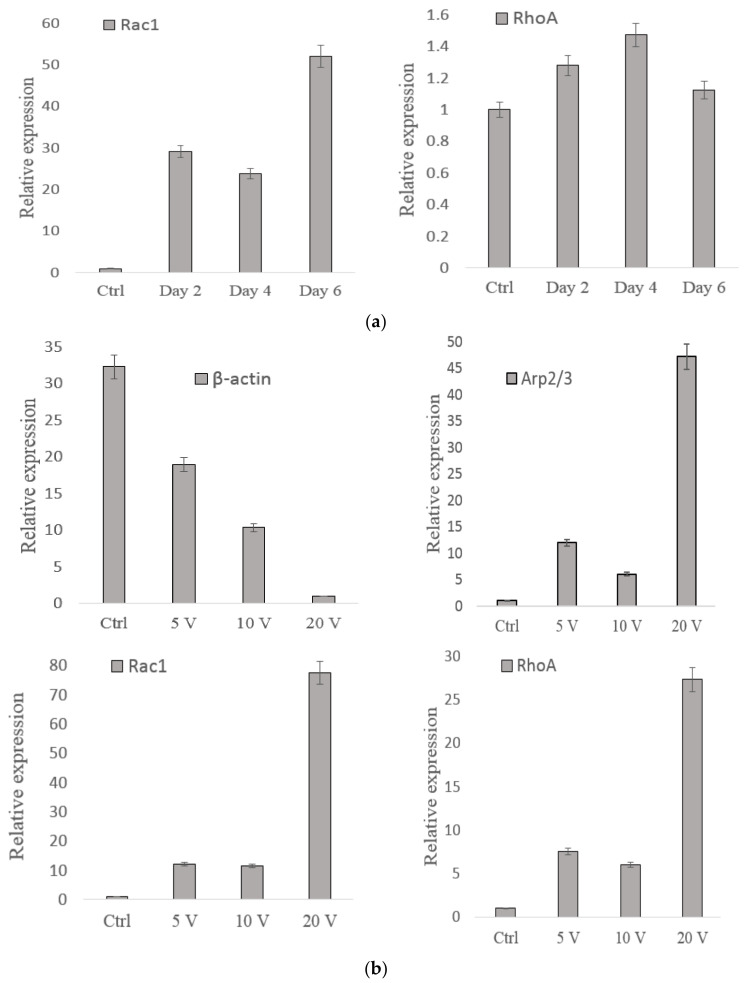
(**a**) Actin-based gene expression results for hMSCs (*p* < 0.01). (**b**). Actin-based gene expression results for NB4 cells (*p* < 0.01).

Figure 6a,b illustrate the actin-based gene expression results for two case studies, where the expression of β-actin and actin-related proteins, such as Arp2/3, and Rho GTPase family (e.g., RhoA and Rac1), were tested. For hMSCs (Figure 6a), compared with the control group, the expression of β-actin, Arp2/3, and Rac1 considerably increased after treatment, while the expression of RhoA did not increase. The β-actin expression increased from days 0 to 6. The Rac1 and Arp2/3 expression levels also generally increased from days 0 to 6, but the Rac1 expression slightly decreased on day 4. The RhoA expression exhibited no obvious change.

For NB4 cells (Figure 6b), the gene expression of RhoA considerably increased after treatment compared with the control group. The β-actin expression decreased as the applied voltage increased. The Rac1 expression increased from 0 V to 5 V, remained unchanged from 5 V to 10 V, and remarkably increased from 10 V to 20 V. The expression levels of both RhoA and Arp2/3 increased and decreased when the voltage increased from 0 V to 10 V. These expression levels sharply increased when the voltage increased from 10 V to 20 V.

The experimental results revealed that only the β-actin expression changed as the mechanical properties of the cells were altered in both case studies. The increase of β-actin expression in the hMSCs case and the decrease in NB4 cells case were consistent with the change of cell stiffness in OT stretching experiments, where the stiffness of the hMSCs increased during differentiation and decreased during electrical stimulation. The gene expression levels of the other cytoskeleton-related proteins did not obviously change in both case studies. Therefore, the change of the actin cytoskeleton mechanical parameters (extracted from the cell stiffness alteration) and the variation of cell biological properties (represented by the expression of PPARg and Bcl-2) can be linked by the differences in the β-actin expressions. We concluded that β-actin could act as an important factor in quantitatively characterizing the interrelationship between the biological and mechanical properties of single cells.

## 4. Conclusions

In this work, the interrelationship between actin cytoskeleton mechanical and cellular biological behavior was characterized quantitatively, in which β-actin expression was used as a key criterion to determine the mechanical properties of the actin cytoskeleton with regard to cell biological behavior. Changes in the biological properties of hMSCs during adipogenesis and leukemia NB4 cell lines during electric field stimulation were particularly investigated. The actin cytoskeleton microstructural model was utilized to quantify the role of the actin cytoskeleton in cell mechanical behavior. The deformation of cells stretched by OT was measured. Results showed that the hMSC-derived adipocytes stiffened as the adipocyte differentiation time was prolonged and the NB4 cells softened as the applied electric stimulation voltage was increased. The experimental data were fitted into the actin cytoskeleton model to exact the actin cytoskeleton mechanical parameters, which implied the F-actin number and density increased with increasing cell stiffness. Gene expression was measured for a better understanding of this phenomenon. Proteins (β-actin, Arp2/3, RhoA, and Rac1) related to actin cytoskeleton mechanics were tested using RT-PCR. For hMSCs, the expression of β-actin generally increased as adipocyte differentiation was enhanced. For NB4 cells, the expression of β-actin decreased as the electric stimulation voltage was increased. These findings agree with the cell stretching results, suggesting that β-actin is the main indicator of changes in cell biological properties related to variations in actin cytoskeleton mechanical properties. This finding reveals how the actin cytoskeleton remodeling profile and the actin-based gene expression reflect the quantitative interrelationship between cell mechanobiological properties.

We are looking forward to obtaining more indicators like β-actin in the following research for a more accurate and thorough characterization of the mechanobiological interrelationship of single cells. This investigation will lead to further exploration of using a particular “biomarker” to evaluate the targeted external treatment of diseased cells in future clinical applications, like early-stage cancer diagnosis and treatment.

## Figures and Tables

**Table 1:** Extracted structural parameters of the actin cytoskeleton of hMSCs during adipogenic differentiation.

F-Actin Structural Parameter	Control	Day 2	Day 4	Day 6
External radius (R_ex_)	8.96 μm	8.89 μm	8.98 μm	9.27 μm
Internal radius (R_in_)	6.02 μm	6.13 μm	6.34 μm	6.75 μm
F-actin density (C_AF_)	22.1 μM	28.6 μM	33.6 μM	36.3 μM
Crosslink density ratio (R)	0.02	0.02	0.02	0.02
Number of F-actin in the network (N_A_)	28,876	33,928	39,611	44,594
Persistence length (L_p_)	3 μm	3 μm	3 μm	3 μm

**Table 2:** Structural parameter estimation of the actin cytoskeleton of NB4 cells treated by electric field stimulation extracted based on the model. Updated data from [21].

F-actin Structural Parameter	Control	Day 2	Day 4	Day 6
External radius (R_ex_)	7.33 μm	7.51 μm	7.45 μm	7.61 μm
Internal radius (R_in_)	6.72 μm	6.88 μm	6.90 μm	6.92 μm
F-actin density (C_AF_)	74.6 μM	74.1 μM	71.2 μM	70.8 μM
Crosslink density ratio (R)	0.02	0.02	0.02	0.02
Number of F-actin in the network (N_A_)	87,656	83,231	78,943	72,881
Persistence length (L_p_)	3 μm	3 μm	3 μm	3 μm

## Data Availability

The data that support the findings of this study are available from the first author, [Ying Li], upon reasonable request.

## References

[1] Zhang R., Lee D.M., Jimah J.R., Gerassimov N., Yang C., Kim S., Luvsanjav D., Winkelman J., Mettlen M., Abrams M.E. (2022). Dynamin regulates the dynamics and mechanical strength of the actin cytoskeleton as a multifilament actin-bundling protein. Nat. Cell Biol..

[2] Dogterom M., Koenderink G.H. (2019). Actin–microtubule crosstalk in cell biology. Nat. Rev. Mol. Cell Biol..

[3] Banerjee S., Gardel M.L., Schwarz U.S. (2020). The actin cytoskeleton as an active adaptive material. Annu. Rev. Condens. Matter Phys..

[4] Ménasché G., Longé C., Bratti M., Blank U. (2021). Cytoskeletal transport, reorganization, and fusion regulation in mast cell-stimulus secretion coupling. Front. Cell Dev. Biol..

[5] Wang Y., Gong J., Yao Y. (2020). Extracellular nanofiber-orchestrated cytoskeletal reorganization and mediated directional migration of cancer cells. Nanoscale.

[6] Uetaki M., Onishi N., Oki Y., Shimizu T., Sugihara E., Sampetrean O., Watanabe T., Yanagi H., Suda K., Fujii H. (2022). Regulatory roles of fibronectin and integrin α5 in reorganization of the actin cytoskeleton and completion of adipogenesis. Mol. Biol. Cell.

[7] Fan Y.L., Zhao H.C., Li B., Zhao Z.L., Feng X.Q. (2019). Mechanical roles of F-actin in the differentiation of stem cells: A review. ACS Biomater. Sci. Eng..

[8] Mo W., Wu J., Qiu Q., Zhang F., Luo H., Xu N., Zhu W., Liang M. (2020). Platelet-rich plasma inhibits osteoblast apoptosis and actin cytoskeleton disruption induced by gingipains through upregulating integrin β1. Cell Biol. Int..

[9] Titushkin I., Cho M. (2009). Regulation of cell cytoskeleton and membrane mechanics by electric field: Role of linker proteins. Biophys. J..

[10] Azadi S., Tafazzoli-Shadpour M., Soleimani M., Warkiani M.E. (2019). Modulating cancer cell mechanics and actin cytoskeleton structure by chemical and mechanical stimulations. J. Biomed. Mater. Res. Part A.

[11] Mack C.P., Somlyo A.V., Hautmann M., Somlyo A.P., Owens G.K. (2001). Smooth muscle differentiation marker gene expression is regulated by RhoA-mediated actin polymerization. J. Biol. Chem..

[12] Di Martino J., Mascalchi P., Legros P., Lacomme S., Gontier E., Bioulac-Sage P., Balabaud C., Moreau V., Saltel F. (2019). Actin depolymerization in dedifferentiated liver sinusoidal endothelial cells promotes fenestrae re-formation. Hepatol. Commun..

[13] Izdebska M., Zielińska W., Grzanka D., Gagat M. (2018). The role of actin dynamics and actin-binding proteins expression in epithelial-to-mesenchymal transition and its association with cancer progression and evaluation of possible therapeutic targets. BioMed Res. Int..

[14] Mahuzier A., Shihavuddin A., Fournier C., Lansade P., Faucourt M., Menezes N., Meunier A., Garfa-Traoré M., Carlier M.F., Voituriez R. (2018). Ependymal cilia beating induces an actin network to protect centrioles against shear stress. Nat. Commun..

[15] Lu W., Fang D.N., Li C.Q., Hwang K.C. (1999). Nonlinear electric–mechanical behavior and micromechanics modelling of cferroelectric domain evolution. Acta Mater..

[16] Li X., Kolega J. (2002). Effects of direct current electric fields on cell migration and actin filament distribution in bovine vascular endothelial cells. J. Vasc. Res..

[17] Rassokhin M.A., Pakhomov A.G. (2014). Cellular regulation of extension and retraction of pseudopod-like blebs produced by nanosecond pulsed electric field (nsPEF). Cell Biochem. Biophys..

[18] Hunley C., Uribe D., Marucho M. (2018). A multi-scale approach to describe electrical impulses propagating along actin filaments in both intracellular and in vitro conditions. RSC Adv..

[19] Wang K., Cheng J., Han Cheng S., Sun D. (2013). Probing cell biophysical behavior based on actin cytoskeleton modeling and stretching manipulation with optical tweezers. Appl. Phys. Lett..

[20] Bai G., Li Y., Chu H.K., Wang K., Tan Q., Xiong J., Sun D. (2017). Characterization of biomechanical properties of cells through dielectrophoresis-based cell stretching and actin cytoskeleton modeling. Biomed. Eng. Online.

[21] Li Y., Li J., Huan Z., Hu Y. (2019). Quantitative characterization of mechano-biological interrelationships of single cells. Int. J. Adv. Manuf. Technol..

[22] Perrin B.J., Ervasti J.M. (2010). The actin gene family: Function follows isoform. Cytoskeleton.

[23] Ruan W., Lai M. (2007). Actin, a reliable marker of internal control?. Clin. Chim. Acta.

[24] Yu M., Le S., Efremov A.K., Zeng X., Bershadsky A., Yan J. (2018). Effects of mechanical stimuli on profilin-and formin-mediated actin polymerization. Nano Lett..

[25] Pollard T.D. (2007). Regulation of actin filament assembly by Arp2/3 complex and formins. Annu. Rev. Biophys. Biomol. Struct..

[26] Ridley A.J. (2006). Rho GTPases and actin dynamics in membrane protrusions and vesicle trafficking. Trends Cell Biol..

[27] Guo F., Debidda M., Yang L., Williams D.A., Zheng Y. (2006). Genetic deletion of Rac1 GTPase reveals its critical role in actin stress fiber formation and focal adhesion complex assembly. J. Biol. Chem..

[28] Wang K., Sun D. (2012). Influence of semiflexible structural features of actin cytoskeleton on cell stiffness based on actin microstructural modeling. J. Biomech..

[29] Palmer J.S., Boyce M.C. (2008). Constitutive modeling of the stress–strain behavior of F-actin filament networks. Acta Biomater..

[30] Chowdhury F., Na S., Li D., Poh Y.C., Tanaka T.S., Wang F., Wang N. (2010). Material properties of the cell dictate stress-induced spreading and differentiation in embryonic stem cells. Nat. Mater..

[31] Shao M., Vishvanath L., Busbuso N.C., Hepler C., Shan B., Sharma A.X., Chen S., Yu X., An Y.A., Zhu Y. (2018). De novo adipocyte differentiation from Pdgfrβ+ preadipocytes protects against pathologic visceral adipose expansion in obesity. Nat. Commun..

[32] Knight T., Luedtke D., Edwards H., Taub J.W., Ge Y. (2019). A delicate balance–The BCL-2 family and its role in apoptosis, oncogenesis, and cancer therapeutics. Biochem. Pharmacol..

